# MEMS measurement microphone compatible to P48 amplifiers

**DOI:** 10.1016/j.ohx.2025.e00627

**Published:** 2025-01-30

**Authors:** F. Huber, F. Toth

**Affiliations:** Institute of Mechanics and Mechatronics, TU Wien, Getreidemarkt 9, 1060 Vienna, Austria

**Keywords:** Experimental acoustics, Pressure sensor, Transducer, Phantom power, IEC 61938, Low-cost

## Abstract

In recent years, micro-electromechanical systems (MEMS) microphones have become increasingly popular in consumer-grade products due to their affordability, performance and low manufacturing variability. This paper presents a pre-amplifier designed to interface with these MEMS capsules and IEC 61938 pro-audio devices, offering high-precision audio measurement and recording at a low cost. The design allows the pre-amplifier and capsule to fit into a $\frac{1}{2}$-inch or 12 mm housing, making it a drop-in replacement for commercial measurement microphones. Comprehensive calculations, simulations and measurements of the pre-amplifier demonstrate its excellent performance. Testing of the device, when paired with a commercially available MEMS capsule, in an acoustically treated chamber, further validates its capabilities. The detailed circuit description also facilitates easy adaptation of the pre-amplifier to other MEMS capsules.

## Highlights

• Pre-amplifier optimized for connecting MEMS microphone capsules to phantom-powered pro-audio equipment; cylindrical housing allows drop-in replacement of conventional microphones.
• Low-cost alternative, reducing microphone cost from over 1000 € to about 10 €.
• Paired pre-amplifier and a state-of-the-art MEMS capsule shows low self-noise, low distortion and excellent frequency flatness up to 10 kHz.
• Restrictions in maximum amplitude and frequency persist; ongoing MEMS innovations are expected to gradually remove these limitations.

## Graphical abstract

**Figure:**
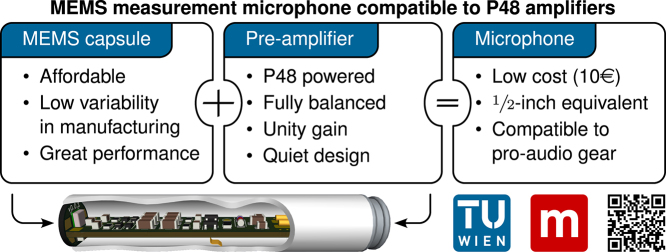

## Specifications table

**Table:** 

**Hardware name**	MEMS Measurement Microphone (mmm)
**Subject area**	Engineering and material sciences

**Hardware type**	Measuring physical properties and in-lab sensors

**Closest commercial analog**	Commercial 1/2-inch pre-polarized condenser measurement microphone

**Open source license**	CC BY 4.0

**Cost of hardware**	8 to 10 €

**Source file repository**	10.17605/osf.io/p5vjt

## 1 Hardware in context

Accurate microphones are crucial for acoustic measurement and recording. Manufacturing the commonly used pre-polarized condenser capsules with the required precision is a tedious process, typically performed by a few specialized companies. Additionally, these capsules require expensive amplifiers and analog to digital converters (ADCs), that often come paired with proprietary software, further increasing the cost of a typical microphone measurement setup. In the past, electret capsules with integrated field effect transistor (FET) were used as cheap alternatives and are still used in pro-audio “measurement microphones”. However, these type of capsules suffer from low manufacturing quality and noise [1].

State-of-the-art MEMS microphones offer a promising solution for these measurement applications. Their automated production process ensures affordability while presenting excellent specifications in terms of noise, sensitivity and linearity [2], [3], [4]. Integrating these capsules into pro-audio hardware results in an affordable measurement chain with an unmatched price-performance ratio. The pre-amplifier in this article is designed to comply with the IEC 61938 [5] analog interface standard P48, ensuring compatibility with a wide range of pro-audio microphone amplifiers and ADCs. Due to higher production volumes, these devices are significantly cheaper than their high-end counterparts but offer similar performance. They use standard audio drivers compatible with many open-source signal processing software packages.

**Fig. 1:**
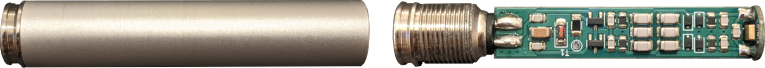
Complete microphone unit (left) and core PCB assembly (right).

To enable drop-in replacement or similar mounting to conventional microphones, a special printed circuit board (PCB) assembly was designed to center the MEMS package at the front face of the cylindrical housing, with a diameter of 12-inch or 12 mm. The pre-amplifier and power conditioning circuitry are encapsulated inside a grounded aluminum tube, alongside the RT3MP Tiny XLR connector, forming a robust yet simple microphone assembly. The complete microphone unit and the PCB core are shown in Fig. 1.

## 2 Hardware description

• The centerpiece of this article is the pre-amplifier, connecting state-of-the-art MEMS capsules to pro-audio equipment complying to the IEC 61938 P48 [5] interface standard. Pairing to the IM73A135 [6] capsule, yields a high performance measurement and recording microphone. Full specifications, alongside details of the circuit design are collected in Section 7 and should be used to adapt the circuit for different MEMS capsules.
• The most significant benefit is the large reduction in cost. Measurement microphone manufacturers typically charge ≈1000 € per microphone. The posed replacement is therefore two orders of magnitude cheaper while offering sufficient performance for most measurement applications.
• Small MEMS capsules (3 × 4 mm) are highly versatile to measure in tight spaces. Instead of using the cylindrical setup, pre-amplifier and capsule can be separated or used on a flexible PCB, to allow acoustic measurements in very tight spaces.
• The main problem with MEMS microphone capsules is their relatively low maximum sound pressure level (SPL) and their resonance. For measurements applications above 135 dB, or with significant amplitudes above 10 kHz, the microphone presented in this article is not recommended [6], [7].

## 3 Design files summary

**Table:** 

Design filename	File type	License	Location of the file
**mechanical**			
mmm.stp	CAD file	CC BY 4.0	10.17605/osf.io/p5vjt
solder_jig.stp	CAD file	CC BY 4.0	10.17605/osf.io/p5vjt
rt3mp_wrench.stp	CAD file	CC BY 4.0	10.17605/osf.io/p5vjt
housing.pdf	Technical drawing	CC BY 4.0	10.17605/osf.io/p5vjt
mmm.pdf	Technical drawing	CC BY 4.0	10.17605/osf.io/p5vjt

**electrical**			

capsule.kicad_pro	EDA file	CC BY 4.0	10.17605/osf.io/p5vjt
capsule.kicad_sch	EDA file	CC BY 4.0	10.17605/osf.io/p5vjt
capsule.kicad_pcb	EDA file	CC BY 4.0	10.17605/osf.io/p5vjt
pcb-conn-4-capsule.kicad_mod	EDA footprint	CC BY 4.0	10.17605/osf.io/p5vjt
pg-llga-5-2.kicad_mod	EDA footprint	CC BY 4.0	10.17605/osf.io/p5vjt
pg-llga-5-2.stp	CAD file	CC BY 4.0	10.17605/osf.io/p5vjt
preamp.kicad_pro	EDA file	CC BY 4.0	10.17605/osf.io/p5vjt
preamp.kicad_sch	EDA file	CC BY 4.0	10.17605/osf.io/p5vjt
preamp.kicad_pcb	EDA file	CC BY 4.0	10.17605/osf.io/p5vjt
pcb-conn-4-preamp.kicad_mod	EDA footprint	CC BY 4.0	10.17605/osf.io/p5vjt
rt3mp.kicad_mod	EDA footprint	CC BY 4.0	10.17605/osf.io/p5vjt
s7181-45r.kicad_mod	EDA footprint	CC BY 4.0	10.17605/osf.io/p5vjt
capsule.wrl	CAD file	CC BY 4.0	10.17605/osf.io/p5vjt
rt3mp.stp	CAD file	CC BY 4.0	10.17605/osf.io/p5vjt
s7181-45r.stp	CAD file	CC BY 4.0	10.17605/osf.io/p5vjt

The full mechanical model is collected in the “mmm.stp” assembly. Additionally, there are the 3D-printed tools “solder_jig.stp”, which is used to align the 90° solder joint between capsule and preamp PCB’s and “rt3mp_wrench.stp”, simplifying tightening and loosing of the RT3MP thread. The metal (aluminum) housing is drawn in “housing.pdf” and the full assemblies outer dimensions and weight are shown in “mmm.pdf”. The electrical design and simulation was conducted in KiCad [8]. The project files, schematics and PCB’s are split into capsule and preamp. To extend KiCad’s standard library, additionally needed footprints and 3D models are provided. To conduct the simulation, SPICE models for the BC860C and BZT55C2V have to be downloaded from the manufacturer’s site.

## 4 Bill of materials summary

**Table:** 

Desig-nator	Component	Nr	Cost/unit - currency	Total cost - currency	Source of materials	Material type
housing	Microphone housing	1	0.533 €	0.533 €	Hardware store	Metal
preamp	Pre-amplifier PCB	1	0.545 €	0.545 €	https://jlcpcb.com/	Composite
capsule	Capsule PCB	1	0.545 €	0.545 €	https://jlcpcb.com/	Composite
RT3MP	Tiny XLR Connector	1	2.090 €	2.090 €	https://mou.sr/45gVZ0l	Composite
MK1	MEMS microphone IM73A135	1	1.330 €	1.330 €	https://mou.sr/3KvBq6O	Semiconductor
C1 to C3	MLCC (NP0) 100 pF 5% 50 V 0805	3	0.021 €	0.063 €	https://mou.sr/4ck5VZq	Ceramic
C4 to C9	MLCC (NP0) 220 nF 5% 50 V 1206	6	0.244 €	1.344 €	https://mou.sr/4cgU661	Ceramic
C10, C11	MLCC (NP0) 270 pF 5% 100 V 0805	2	0.041 €	0.082 €	https://mou.sr/3VEJZT6	Ceramic
C12	MLCC (X7R) 22uF 10% 10 V 1206	1	0.117 €	0.117 €	https://mou.sr/4ac46wP	Ceramic
C13, C14	MLCC (NP0) 6.8 nF 5% 100 V 0805	2	0.050 €	0.100 €	https://mou.sr/4b0abwa	Ceramic
CMK1	MLCC (X7R) 0.1 uF 10% 100 V 0805	1	0.048 €	0.048 €	https://mou.sr/4b6J3vk	Ceramic
D1 to D4	Schottky 100 V 150 mA SOD-323F	4	0.109 €	0.436 €	https://mou.sr/3zgyUz0	Semiconductor
D5	Zener 2.7 V 0.5 W SOD-80	1	0.055 €	0.055 €	https://mou.sr/3XoMQ3U	Semiconductor
N1	EMI Spring Contact	1	0.263 €	0.263 €	https://mou.sr/3xjqg27	Metal
Q1, Q2	BC860C SOT-23	2	0.045 €	0.090 €	https://mou.sr/3Xh1HNT	Semiconductor
R1, R2	Resistor 33k 1% 1/8W 0805	2	0.014 €	0.028 €	https://mou.sr/4ejOMRo	Metal
R3, R4	Resistor 150k 1% 1/8 W 0805	2	0.014 €	0.028 €	https://mou.sr/3z1wDHX	Metal
R5	Resistor 4.7k 1% 1/4 W 1206	1	0.018 €	0.018 €	https://mou.sr/3VlvX7I	Metal

Since the cost of electronic components varies significantly with quantity, the unit price is measured based on an order of 100 pieces. The PCBs cost include a stencil for solder paste application. Additionally, the alignment jig “solder_jig.stp” and the Tiny XLR wrench “rt3mp_wrench.stp” are recommended for assembly (see Section 3). To manufacture the thread for the RT3MP connector, a 716−32 UN tap is required. Consumables like Solder Paste, Solder Wire, etc. are prerequisites for the assembling process.

## 5 Build instructions

**Fig. 2:**
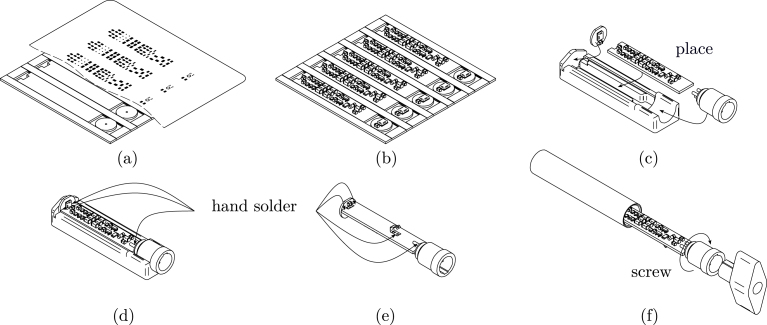
Steps for the assembly process.

This section provides useful information for the assembly process. To ensure a smooth process, please follow the steps outlined below. Additionally, the full process is illustrated in Fig. 2. For manufacturing more than one microphone, it is recommended to arrange multiple PCBs in a panel.

(a) Place the panel on a flat surface. Align and secure the stencil, then apply the solder paste. For this build, we used Chipquik SMD291SNL10 paste. Ensure the stencil remains stationary throughout the procedure. Proceed only if the pads are evenly covered with paste and there is no smearing.
(b) Carefully place the components on the solder pads and press them down gently, to embed their contact surfaces in paste. Use a reflow-oven or a hot-plate to solder the components to the panel, ensuring you follow the reflow profile specified for the solder paste used.
(c) Carefully separate the PCBs at the V-grooves and place them in the 3D-printed solder jig. Double-check the alignment, ensuring the outer ring of the connectors sits flush with the jig and the connector tab is positioned at the top.
(d) While pressing down on the preamp board, solder the two top pads connecting the capsule to the preamp PCB. Be sure to keep the procedure as short as possible. Then solder the two top facing connector pins to the preamp board.
(e) Flip the board around and solder all bottom pads, including the EMI Spring Contact N1 in the shown orientation.
(f) Finally, the PCB assembly can be screwed into the housing. This can be done by hand or with the 3D-printed wrench. Ensure that the capsule PCB sits flush with the other side of the housing.

## 6 Operation instructions

For normal operation the environmental conditions specified for the hardware must be met, with the most critical component typically being the MEMS capsule. For the components used in this article the limiting factor is the capsule, which operates within a temperature range of -40 °C to 85 °C. Although the capsule is dust- and water-resistant, the lack of a seal between the housing and the capsule PCB prevents it from operating under these conditions [6]. However, appropriate sealing methods (such as glue, silicone, or grease) could potentially address this issue, though such solutions are not tested in this study.

For the acoustic interface, it is important to ensure that the maximum pressure rating of the capsule is not exceeded to avoid irreversible damage. The acoustic overload point (AOP) for the IM73A135 is 135 dB. Additionally, it is important to keep the porthole clean, as particles and fluids can obstruct it and are difficult to remove.

The pre-amplifier should be connected to a microphone amplifier compatible with the IEC 61938 standard, adhering to the P48 supply specification. It is not recommended to plug or unplug the device while phantom power is on, although the device includes diodes to protect against voltage spikes that may result from such actions.

## 7 Validation and characterization

### 7.1 Pre-amplifier analytical calculations

**Fig. 3:**
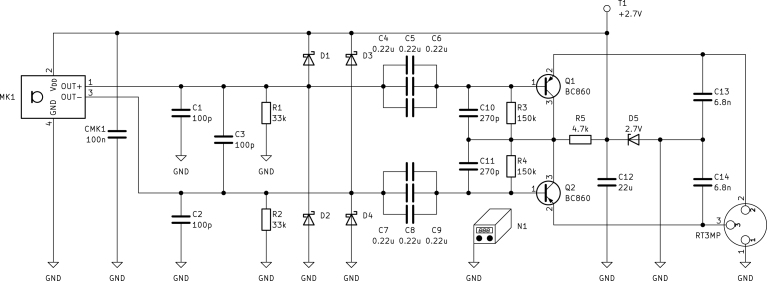
Full pre-amplifier circuit diagram.

During the development of the pre-amplifier circuit, analytical calculations are necessary to ensure the expected performance. To facilitate adaptation of the circuit for different use cases, these calculations are detailed in this section. The complete circuit is shown in Fig. 3. The analytical computations are divided into their DC and small signal part. By exploiting the symmetric design, only the positive (top) signal part is analyzed, with necessary adjustments made for components connecting both halves of the circuit. The operator used for parallel summing resistances and series summing capacitances, is defined as

(1) $$A∥B\overset{!}{=}\frac{1}{\frac{1}{A}+\frac{1}{B}}\;\text{.}$$

To properly dimension the pre-amplifier, the capsule’s maximum output signal must be known. This is calculated according to the capsule manufacturer’s implementation guide [9]. The peak amplitude of the differential output voltage is reached at the acoustic overload point (AOP) and can be calculated as

(2) $$\max\left(u_{\text{MK1,p}}\right)=10^{\frac{S+\mathrm{AOP}-94\;dB+3\;dB}{20\;dB}}=2\;V_{\text{p}}\;\text{,}$$

with the capsule sensitivity S=−38dBV and AOP=135dB [9].

#### DC model.

**Fig. 4:**
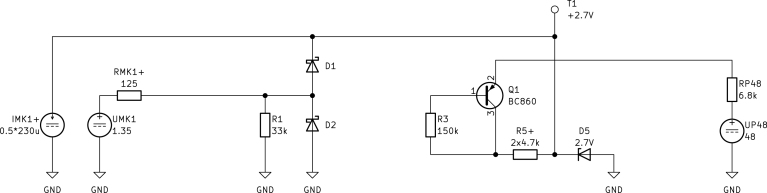
Top (positive) half of the pre-amplifier’s DC model.

To obtain the DC model, all capacitors are replaced with open circuits. The capsule MK1 is substituted by a constant current source $I_{\text{MK1}}=230\;µA$ on the power supply rail, a voltage source $U_{\text{MK1}}=1.35\;V$ and a differential output resistance $R_{\text{MK1}}=250\;\Omega$ at the outputs. These values are taken from the capsule’s datasheet [6]. Due to the symmetric design, the value of $R_{5}$ (in Ω) must be doubled and $I_{\text{MK1}}$ (in A) and $R_{\text{MK1}}$ (in Ω) halved to maintain correct results; the adjusted components are indicated with the subscript “+”. Connected to RT3MP of the full model are $R_{\text{P48}}$ (in Ω) and $U_{\text{P48}}$ (in V) according to the P48 phantom power specifications [5]. The simplified DC model is drawn in Fig. 4.

Starting from the phantom power supply $U_{\text{P48}}$ we can calculate $Q_{1}$’s emitter current $I_{\text{E,Q1}}$ (in A) by considering the right mesh

(3) $$U_{\text{P48}}=R_{\text{P48}}I_{\text{E,Q1}}-U_{\text{BE}}+R_{3}I_{\text{B,Q1}}+R_{\text{5+}}\left(I_{\text{C,Q1}}-I_{\text{B,Q1}}\right)+U_{\text{D5}}\;\text{,}$$

with the phantom power supply resistance $R_{\text{P48}}$ (in Ω), $Q_{1}$’s collector and base currents $I_{\text{C,Q1}}$ and $I_{\text{B,Q1}}$ (in A) and the resistances $R_{3}$, $R_{5}$ (in Ω) alongside the Zener diode’s voltage drop $U_{\text{D5}}$ (in V).

With the identities $I_{\text{C,Q1}}=I_{\text{E,Q1}}+I_{\text{B,Q1}}$, $I_{\text{B,Q1}}h_{\text{FE}}=I_{\text{C,Q1}}$ and by inserting the datasheet’s [10] typical values for the base emitter voltage $U_{\text{BE}}=-0.65\;V$ and the DC current gain $h_{\text{FE}}=610$, Eq. (3) can be rearranged to

(4) $$I_{\text{C,Q1}}=\frac{U_{\text{P48}}+U_{\text{BE}}-U_{\text{D5}}}{\left(R_{\text{P48}}+R_{\text{5+}}\right)\left(1-\frac{1}{h_{\text{FE}}}\right)+R_{3}\frac{1}{h_{\text{FE}}}}=2.719\;mA\;\;\text{and}\;\;I_{\text{E,Q1}}=I_{\text{C,Q1}}\left(1-\frac{1}{h_{\text{FE}}}\right)=2.715\;mA\;\text{.}$$

The collector resistor $R_{5}$ is dimensioned in a way, to achieve $\approx U_{\text{P48}}/2$ at $Q_{1}$’s base terminal. The resistor $R_{3}$ should be adjusted accordingly, to provide at least ten times the needed base current, so $10R_{3}/R_{5}\ll h_{\text{FE}}$. The computed currents in Eq. (4) can be used to check the current of the Zener diode D5. By again considering the symmetry we can compute

(5) $$I_{\text{D5}}=2I_{\text{E,Q1}}-2I_{\text{MK1+}}=5.200\;mA\;\text{,}$$

closely matching the recommended test current of 5 mA taken from the datasheet [11] and therefore, ensuring good voltage stability. Additionally, we have to check the components for power dissipation against their datasheet values [10], [11]

(6) $$P_{\text{D5}}=U_{\text{D5}}I_{\text{D5}}=0.014\;W\overset{!}{\ll }P_{\text{D5,max}}=0.5\;W\;\text{,}$$

(7) $$P_{\text{R5}}=R_{5}I_{\text{D5}}^{2}=0.14\;W\overset{!}{\ll }P_{\text{R5,max}}=0.25\;W\;\;\text{and}$$

(8) $$P_{\text{Q1}}=\left(U_{\text{P48}}-\left(R_{\text{P48}}+R_{\text{5+}}\right)I_{\text{E,Q1}}-U_{\text{D5}}\right)I_{\text{C,Q1}}=0.0036\;W\overset{!}{\ll }P_{\text{Q1,max}}=0.25\;W\;\text{.}$$

Examining the left mesh, the Schottky diodes $D_{1}$ and $D_{2}$ provide protection for MK1 during plugging and unplugging events, where the capacitor banks $C_{4}$ to $C_{9}$ are charged or discharged. The output is clipped based on the forward voltage of the diodes. According to the datasheet [12] of the selected BAT46WS diodes, their forward voltage at low currents is $U_{\text{D1,F}}=U_{\text{D1,F}}=0.25\;V$. Therefore, the maximum signal amplitude before clipping can be calculated as

(9) $$u_{\text{MK1,p,clipping}}=2\left(U_{\text{MK1}}-U_{\text{D1,F}}\right)=2.2\;V\overset{!}{>}\max\left(u_{\text{MK1,p}}\right)\;\text{,}$$

with the factor two due to the symmetric design. In theory no distortion due to the protection diodes is expected, referring to Section 7 for the corresponding distortion measurements. When selecting the diodes, it is important to chose their reverse voltage above $U_{\text{P48}}$ (in V). The microphone tested in this paper uses BAT46WS diodes with a reverse voltage of 100 V [12]. Leakage currents are negligible small and the maximum current is limited by $R_{\text{P48}}$.

When adapting this circuit for different supply voltages (e.g. P12, P24, …) the following steps are necessary:

• Neglect the voltage drop of $R_{3}$.
• Select $R_{5}$ to achieve a Zener current $I_{\text{D5}}$ of 5 mA.
• Select $R_{5}$ to achieve $U_{\text{B,Q1}}\approx U_{\text{P48}}/2$
• Consider the voltage drop of $R_{3}$.
• Adjust $R_{3}$ to meet the ratio $10R_{3}/R_{5}\gg h_{\text{FE}}$.
• Check $I_{\text{D5}}$ and $U_{\text{B,Q1}}$ with considered $R_{3}$.

#### Small signal (AC) model.

**Fig. 5:**
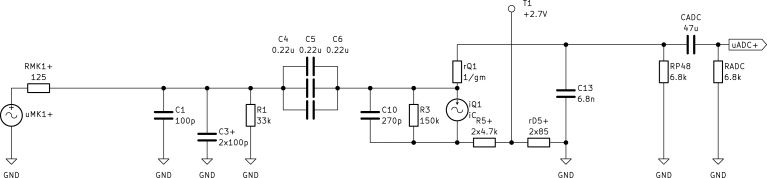
Top (positive) half of the pre-amplifier’s small signal (AC) model.

The small signal model is obtained by shorting DC voltage sources and opening DC current sources. The stabilization capacitors $C_{\text{MK1}}$ and $C_{12}$ are neglected and components adjusted for symmetry are again marked with the subscript “+”. Fig. 5 illustrates the small signal model, with RT3MP connected to a microphone amplifier complying with the P48 phantom power specifications [5].

The capsule MK1 is modeled as a voltage source with an amplitude of $u_{\text{MK1+}}=\max\left(u_{\text{MK1,p}}\right)/2$ (in V), computed in Eq. (2), and a series output resistance of $R_{\text{MK1+}}=R_{\text{MK1}}/2$ (in Ω). Both values are halved due to the differential output signal and are derived from the datasheet [6] specifications. Transistor $Q_{1}$ and Zener diode $D_{5}$ are replaced with their respective small signal models. The small signal resistance of $Q_{1}$ is defined as the dual of its transadmittance calculated as

(10) $$r_{\text{Q1}}=\frac{1}{g_{\text{m}}}=\frac{U_{\text{T}}}{I_{\text{C,Q1}}}=9.2\;\Omega \;\text{,}$$

with the thermal voltage $U_{\text{T}}=kT/q\approx 25\;mV$ and the DC collector current from Eq. (4). The small signal Zener resistance $r_{\text{D5}}=85\;\Omega$ is taken from the datasheet [11] for a test current of 5 mA, which closely matches $I_{\text{D5}}$ calculated in Eq. (5). The Zener resistance $r_{\text{D5}}$ (in Ω), along with $R_{5}$ (in Ω) and the filter capacitor $C_{3}$ (in F) are doubled due to symmetry and marked as $r_{\text{D5+}}$, $R_{\text{5+}}$ and $C_{\text{3+}}$ respectively. In the small signal model the ADC’s input resistance $R_{\text{ADC}}$ (in Ω) must be considered. For the tested systems (RME Fireface UC, RME Fireface UFX+), ADC resistance was approximately the same as $R_{\text{P48}}$. This probably varies between manufacturers, as it is not defined in IEC 61938 [5]. While the effect is small, for precise measurement, the ADC’s input resistance should be determined and considered.

The output voltage of the pre-amplifier is measured as voltage drop across the ADC’s input resistor $R_{\text{ADC}}$. Because only half of the circuit is considered, we can only analyze the positive part $u_{\text{ADC+}}$ (in V) of the ADC’s input voltage. To calculate the AC gain v, the filter capacitors $C_{1}$, $C_{\text{3+}}$, $C_{10}$ and C13 are opened while coupling capacitors $C_{4}$ to $C_{6}$ and $C_{\text{ADC}}$ are replaced with short circuits. With the assumption of a small base current $\ll i_{\text{B,Q1}}$ and bias current $\ll i_{\text{R3}}$, the pre-amplifier gain can be expressed as

(11) $$v=\frac{u_{\text{ADC+}}}{u_{\text{MK1+}}}=\frac{R_{1}}{R_{\text{MK1}}+R_{1}}\frac{R_{\text{P48}}∥R_{\text{ADC}}}{r_{\text{Q1}}+R_{\text{P48}}∥R_{\text{ADC}}}=0.9935\;\;\text{or}\;\;-0.056\;dB\;\text{.}$$

Because of the emitter follower (common collector) configuration, a gain close to unity is expected.

#### Impedance and loads.

The small signal model in Fig. 5 can also be used to compute input and output impedances, both are split into resistive and capacitive part. The differential input resistance $r_{\text{in}}$ (in Ω) is computed by

(12) $$r_{\text{in}}=2\left(R_{1}∥\left(h_{\text{FE}}-1\right)\left[r_{\text{Q1}}+\left(R_{\text{P48}}∥R_{\text{ADC}}\right)\right]∥\left(R_{3}+R_{\text{5+}}+r_{\text{D5+}}\right)\right)=54\;k\Omega \;\text{.}$$

The factor 2 arises due to symmetry and the individual terminal impedance of $r_{\text{in}}/2$ closely matches the recommended values in the capsule’s datasheet [6]. The differential input capacitance $C_{\text{in}}$ (in F) is calculated with

(13) $$C_{\text{in}}=\frac{1}{2}\left(C_{1}+C_{\text{3+}}+\frac{1}{C_{4}+C_{5}+C_{6}}∥\left(C_{\text{10}}+\frac{C_{13}+C_{\text{ADC}}}{h_{\text{FE}}-1}\right)\right)=35\;nF\;\text{.}$$

According to Eqs. (12), (13), the differential input impedance depends on the load values $R_{\text{ADC}}$ (in Ω) and $C_{\text{ADC}}$ (in F), which are not standardized and have to be considered for each used ADC individually. To calculate the output impedance, the load ($R_{\text{P48}}$, $C_{\text{ADC}}$, $R_{\text{ADC}}$) is disconnected. The differential output resistance $r_{\text{out}}$ (in Ω) is calculated by

(14) $$r_{\text{out}}=2\left(r_{\text{Q1}}+\frac{R_{\text{MK1}}∥R_{1}∥\left(R_{3}+R_{\text{5+}}+r_{\text{D5+}}\right)}{h_{\text{FE}}-1}\right)=18.6\;\Omega \;\text{.}$$

Analogously, the differential output capacitance $C_{\text{out}}$ (in F) is computed with

(15) $$C_{\text{out}}=\frac{1}{2}\left(C_{13}+\frac{1}{h_{\text{FE}}-1}\left(C_{10}+\left(C_{4}+C_{5}+C_{6}\right)∥\left(C1+C_{\text{3+}}\right)\right)\right)=3.4\;nF\;\text{.}$$

#### Filters.

Once more, the small signal model can be used to characterize the filter capacitors in the circuit, allowing for analysis of the frequency dependent behavior of the pre-amplifier. Since typically available ADC’s sample at rates up to 384 kHz, the highest frequency of interest is 192 kHz. To ensure flat behavior up to this frequency, the lowpass cutoff frequency is chosen to be ten times higher, around 2 MHz. The highpass cutoff frequency depends on the AC coupling of the ADC, the capsule used and space limitations for large coupling capacitors. The lowest frequency is therefore set at the limit of the audible range 16 Hz with a cutoff frequency ten times lower, around 1.6 Hz.

Following the signal path, the first filter is a lowpass consisting of the capsules output impedance $R_{\text{MK1}}$ (in Ω) and $C_{1}+C_{\text{3+}}$ (in F) [6]. The cutoff frequency can be calculated as

(16) $$f_{\text{LP1}}=\frac{1}{2\pi R_{\text{MK1+}}\left(C_{1}+C_{\text{3+}}\right)}=4.244\;MHz\;\text{.}$$

The capacitor bank $C_{4}$ to $C_{6}$ (in F), paired with the resistive impedance of the following circuit, acts as a highpass filter. The cutoff frequency is calculated with

(17) $$f_{\text{HP1}}=\frac{1}{2\pi \left[\left(h_{\text{FE}}-1\right)\left(r_{\text{Q1}}+\left(R_{\text{P48}}∥R_{\text{ADC}}\right)\right)∥\left(R_{3}+R_{\text{5+}}+r_{\text{D5+}}\right)\right]\left(C_{4}+C_{5}+C_{6}\right)}=1.627\;Hz\;\text{,}$$

where the resistive impedance is calculated similarly to Eq. (12). $C_{10}$ (in F) paired with the input impedance act as lowpass filter, with a cutoff frequency of

(18) $$f_{\text{LP2}}=\frac{1}{2\pi R_{\text{MK1}}C_{10}}=4.716\;MHz\;\text{.}$$

After amplification the output resistance of Q1, calculated in Eq. (14), forms the third lowpass filter in combination with $C_{13}$ (in F). The cutoff frequency results to

(19) $$f_{\text{LP3}}=\frac{1}{\pi r_{\text{out}}C_{13}}=2.517\;MHz\;\text{.}$$

Finally, the highpass behavior of the connected ADC converter is considered. For the given values, the cutoff frequency is calculated with

(20) $$f_{\text{HP2}}=\frac{1}{2\pi R_{\text{ADC}}C_{\text{ADC}}}=0.498\;Hz\;\text{.}$$

Note, that for different ADC’s $f_{\text{HP2}}$ may differ.

The small signal gain found in Eq. (11) and the filter frequencies of this section can be used to assemble the analytical transfer function of the system

(21) $$\overset{˜}{G}\left(f\right)=\frac{v\left(i\frac{f}{f_{\text{HP1}}}\right)\left(i\frac{f}{f_{\text{HP2}}}\right)}{\left(1+i\frac{f}{f_{\text{LP1}}}\right)\left(1+i\frac{f}{f_{\text{LP2}}}\right)\left(1+i\frac{f}{f_{\text{LP3}}}\right)\left(1+i\frac{f}{f_{\text{HP1}}}\right)\left(1+i\frac{f}{f_{\text{HP2}}}\right)}\;\text{,}$$

used to compare analytical, simulated and measured frequency responses in Fig. 7.

### 7.2 Pre-amplifier simulation in SPICE

**Fig. 6:**
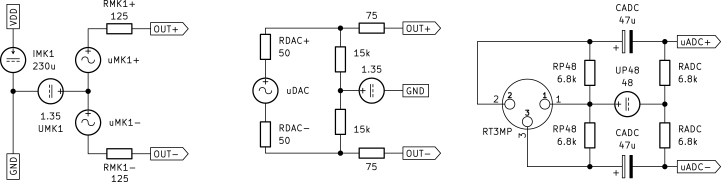
Simulation model of the IM73A135 capsule (left), Fireface UFX+ audio interface’s DAC used as capsule replacement (center) and its ADC circuit including P48 power used for simulation and measurement (right).

To conduct a simulation of the circuit shown in Fig. 3, input (capsule) and output (audio interface) of the pre-amplifier have to be modeled accordingly. Both simplified models are shown in Fig. 6. The capsule uses the DC voltage and current given in the datasheet [6], while the audio interface is modeled after the exemplary circuit in IEC 61938. The values of $R_{\text{ADC}}$ and $C_{\text{ADC}}$ are measured from the specific audio interface at hand. The internal SPICE simulator of KiCad [8] is used to conduct harmonic analysis of the circuit. While $u_{\text{MK1+}}=u_{\text{MK1-}}=\max\left(u_{\text{MK1,p}}\right)/2$ (in V), obtained in Eq. (2), are used as input sources, the output voltages $u_{\text{ADC+}}$ and $u_{\text{ADC-}}$ (in V) are measured at their respective tags. Except for the models $Q_{1}$, $Q_{2}$ and $D_{5}$, standard SPICE models are used. Besides the simulation file, the required models for the mentioned parts are explained in Section 3.

### 7.3 Measurement

To verify the results of the previous chapters, a series of measurements is conducted. For all of them, the RT3MP connector of the pre-amplifier is connected to the Fireface UFX+ microphone input, used for ADC conversion, while the digital to analog converter (DAC) outputs are used for signal generation if needed. The ADC’s internal circuitry is shown in Fig. 6. The measurement routines are custom Python scripts using the ASMU package [13]. To switch between time- and frequency-domain the real valued fast Fourier transform algorithm implemented in NumPy [14] is used. To reduce edge-artifacts the time signals are pre-filtered with a 1 s hanning window, shifted by 0.5 s. The transformed signals are then processed and averaged, or averaged directly. The typical measurement duration is 60 s.

#### Pre-amplifier.

**Fig. 7:**
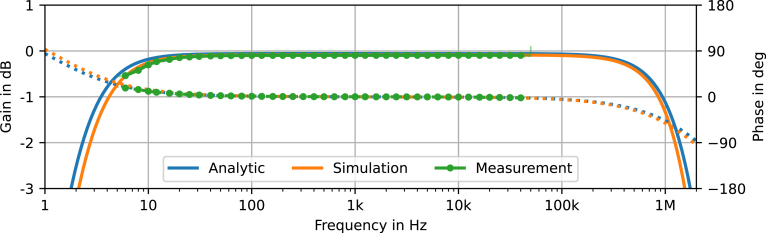
Comparison of analytically calculated, simulated and measured frequency response in magnitude (solid) and phase (dotted). The light colored green line shows the unfiltered measurement result, one-third octave band averages are indicated with dots. (For interpretation of the references to color in this figure legend, the reader is referred to the web version of this article.)

As a first step, the isolated pre-amplifier is measured, solidifying the previous results as a solid MEMS capsule interface to the P48 standard. To measure the frequency response, the capsule is replaced by 75 Ω resistors on each balanced line and a DC source fed over 15 kΩ resistors, to center the incoming AC voltage between ground and supply rail. The equivalent circuit is drawn in Fig. 6 (center), which shows the impedance match to the capsule resistance of $R_{\text{MK1}}=250\;\Omega$. This setup is connected to the Fireface UFX+ balanced line output; additionally to the mentioned connection between the pre-amplifier’s output and the audio interface’s microphone input. The audio interface is used to generate a pink noise signal with a maximum amplitude of $u_{\text{DAC}}=2\;V_{\text{p}}$ or 3 dBV at the line output, equivalent to the AOP of the IM73A135 [6] capsule, as shown in Eq. (2). Simultaneously, the generated signal is recorded alongside the pre-amplifiers output. The two recordings, of output and input, are then used to compute the transfer function. The signals are transformed to the frequency domain and divided for each window position to yield the transfer functions, which are then averaged to retrieve the final result. Magnitude and phase are plotted in Fig. 7 and compared to the analytical and simulation results, presenting excellent agreement.

**Fig. 8:**
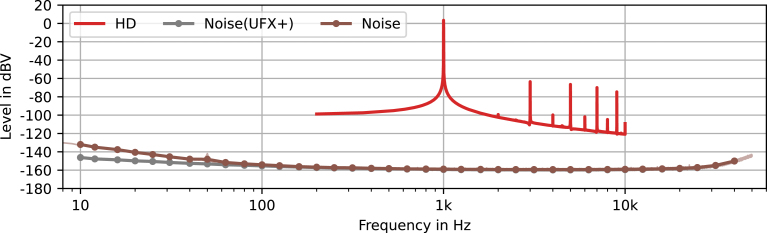
Harmonic distortion (HD) and noise magnitude of the pre-amplifier and the audio interface (UFX+). The light colored brown line shows the unfiltered measurement result, one-third octave band averages are indicated with dots. (For interpretation of the references to color in this figure legend, the reader is referred to the web version of this article.)

Continuing with the pre-amplifier, harmonic distortion (HD) performance is evaluated. Therefore, the capsule is again substituted by the circuit in Fig. 6 and connected to the audio interface’s balanced line output. For the harmonic distortion measurement a sine wave with a frequency of $f_{\text{HD}}=1\;kHz$ and peak amplitude of $u_{\text{DAC}}=2\;V_{\text{p}}$ or 3 dBV is generated at the pre-amplifiers input. Again the generated signal and the output are recorded. The transfer function is computed in the same way as for the frequency response measurement, the resulting harmonics are shown in Fig. 8.

The total harmonic distortion (THD) is calculated as the square root of the sum of the squares of the first H harmonic’s amplitudes divided by the root frequency’s amplitude [15] as

(22) $${\mathrm{THD}}_{\left(H\right)}=\frac{1}{\left|\overset{˜}{p}\left(f_{\text{HD}}\right)\right|}\sqrt{\overset{H}{\underset{h=2}{\sum }}\overset{˜}{p}{\left(hf_{\text{HD}}\right)}^{2}}\;\text{with}\;\;H=5\;\;\text{we obtain}$$

(23) $${\mathrm{THD}}_{\left(5\right)}=0.0552\;\text{\%}\;\;\text{or}\;\;-65.2\;dB\;\;\text{at}\;\;2\;V_{\text{p}}\;\;\text{or}\;\;3\;dB\text{V}\;\;\text{and}$$

(24) $${\mathrm{THD}}_{\left(5\right)}=0.0013\;\text{\%}\;\;\text{or}\;\;-97.5\;dB\;\;\text{at}\;\;1\;V_{\text{p}}\;\;\text{or}\;\;-3\;dB\text{V}\;\text{.}$$

Note, that for the selected signal amplitude the harmonic distortion is affected by the input protection diodes $D_{1}$ to $D_{4}$. If the amplitude is reduced by 6 dB, the harmonic distortion decreases significantly.

**Fig. 9:**
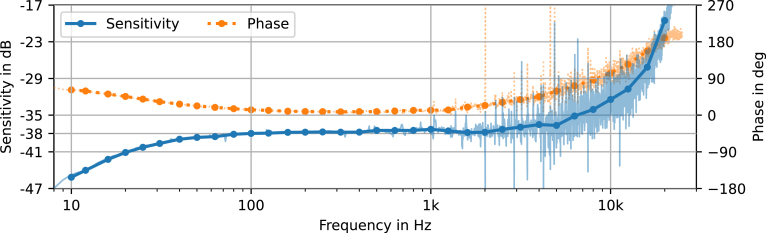
Frequency dependence of the microphone sensitivity in magnitude (solid) and phase (dotted). Light colored lines (blue, orange) show the unfiltered measurement results, one-third octave band averages are indicated with dots. (For interpretation of the references to color in this figure legend, the reader is referred to the web version of this article.)

To measure the self-noise, the pre-amplifier inputs are shorted. The output is recorded and transformed to the frequency domain, the results for each window position are averaged directly, which discards the phase information. The harmonic content of the noise is plotted in Fig. 8, with the points indicating one-third octave band averages. Additionally, we evaluated the self noise of the interface, by shorting the used microphone input and conducting the same measurement. The noise root-mean-square (RMS) value is computed for a frequency range of 20 Hz to 20 kHz from the harmonic amplitude spectrum $\overset{˜}{p}\left(f\right)$ as

(25) $${\mathrm{Noise}}_{\left(w\right)}=\sqrt{\int_{20\;Hz}^{20\;kHz}{\left[R_{w}\left(f\right)\overset{˜}{p}\left(f\right)\right]}^{2}\;df}\;\;\text{with}\;\;w\in \left[\text{lin.},\text{A}\right]\;\;\text{we obtain}$$

(26) $${\mathrm{Noise}}_{\text{(lin.)}}=1.6596\;{µV}_{\left(\mathrm{RMS}\right)}\;\;\text{or}\;\;-115.60\;dB\text{V}\;\;\text{and}$$

(27) $${\mathrm{Noise}}_{\text{(A)}}=1.2455\;{µV}_{\left(\mathrm{RMS}\right)}\;\;\text{or}\;\;-118.09\;dB\text{V(A)}\;\text{,}$$

with the weighting functions $R_{\text{lin.}}=1$ and $R_{\text{A}}\left(f\right)$ as defined in IEC 61672-1 [16]. Fig. 8 shows that, except for low frequencies, the noise of the pre-amplifier is indistinguishable from the audio interface noise.

#### Microphone.

To obtain a full measurement microphone, the pre-amplifier is combined with the IM73A135 [6] capsule. As acoustic reference, a Brüel & Kjær measurement system is used. This reference system consists of a Type 4190 [7] free-field capsule, a Type 2669 [17] pre-amplifier and a Type 2690 [18] microphone conditioning amplifier. The line output of the conditioning amplifier is connected to a microphone input of the Fireface UFX+, the same type of input used to connect the pre-amplifier introduced in this article. The Brüel & Kjær reference setup is calibrated with a Type 4231 [19] sound calibrator. The assembled MEMS microphone and the reference microphone are mounted closely together, with both membranes facing in the same direction (0° incident angle) and aligned on the same plane. The assembly is then placed into the center of an acoustically treated box, with a Genelec 8020C [20] speaker placed at a distance of 1 m facing the microphones. The speaker is connected to the audio interface’s line output and calibrated with the reference system, to produce prescribed SPLs.

To measure the sensitivity, the audio interface is used to generate a pink noise signal with an average SPL of 94 dB at the capsule, while simultaneously recording the signal of the reference system alongside the pre-amplifiers output. Computing the transfer function between the voltage calibrated MEMS microphone input and the pressure calibrated reference input yields the free-field sensitivity of the pre-amplifier and capsule under test. This procedure is based on the recommendations in IEC 60268-4 [21] and IEC 61094-5 [22]. The obtained sensitivity is plotted over frequency in Fig. 9. The same setup was used by Djurek at al. [23] and a similar setup, but with facing capsule membranes, was implemented by Prato et al. [24]. The second setup has the advantage of reducing the reflection surface by utilizing the microphone bodies as wave guides, but it computes the transfer function between 0° and 180° incident angle, which we want to avoid. The transfer function above 4 kHz experiences increased noise, probably caused by the reflections of microphone bodies and mounting fixtures disturbing the local acoustic field. The strong rise above 10 kHz can be explained by the resonance frequency and is also documented in the capsule’s datasheet [6]. An evaluation at 1 kHz yields the 0° incident free-field sensitivity of the microphone under test

(28) $$S_{1\;kHz}=13.62\;mV\;{Pa}^{-1}\;\;\text{or}\;\;-37.3\;dB\;\text{,}$$

with the latter referenced to 1 V Pa^-1^. The sensitivity matches the value given in the datasheet [6].

**Fig. 10:**
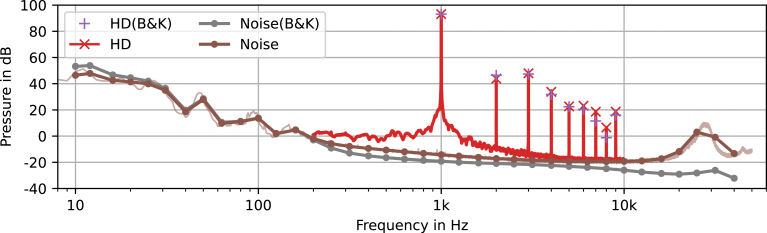
Harmonic distortion (HD) and noise magnitude of pre-amplifier with IM73A135 and the (B&K) reference system. The light colored brown line shows the unfiltered measurement results, one-third octave band averages are indicated with dots. (For interpretation of the references to color in this figure legend, the reader is referred to the web version of this article.)

Harmonic distortion is measured based on IEC 60268-4 [21]. The arrangement stays unchanged, but a 94 dB sine wave is used as excitation signal. The harmonic response is shown in Fig. 10 and the THD, calculated with Eq. (22) yields

(29) $${\mathrm{THD}}_{\left(5\right)}=0.66\;\text{\%}\;\;\text{or}\;\;-43.6\;dB\;\text{.}$$

Note, that the THD measured with the reference system shows similar values, pointing to the speaker as main contributor of distortion. The pre-amplifier distortion at high SPL, investigated in Eqs. (23), (24), is negligibly small compared to the source distortion and the expected distortion of the capsule [6].

The noise measurement uses the same setup, but with a turned off speaker. The results of the microphone introduced in this article and the reference system are shown in Fig. 10. One can see that the used acoustically treated box shows bad isolation below 200 Hz. Moreover, the capsule’s resonance frequency of ≈30kHz is clearly visible. Therefore, Eq. (25) is facilitated within a range of 200 Hz to 20 kHz, to evaluate the noise performance over its RMS value yielding

(30) $${\mathrm{Noise}}_{\text{(lin.)}}=5.4524\;{µV}_{\left(\mathrm{RMS}\right)}\;\;\text{or}\;\;-105.27\;dB\text{V}\;\;\text{or}\;\;0.4004\;{mPa}_{\left(\mathrm{RMS}\right)}\;\;\text{or}\;\;26.03\;dB\;\;\text{and}$$

(31) $${\mathrm{Noise}}_{\text{(A)}}=3.9583\;{µV}_{\left(\mathrm{RMS}\right)}\;\;\text{or}\;\;-108.05\;dB\text{V(A)}\;\;\text{or}\;\;0.2907\;{mPa}_{\left(\mathrm{RMS}\right)}\;\;\text{or}\;\;23.25\;dB\text{(A)}\;\text{.}$$

The emphasized difference between linear and A-weighted results comes from the pronounced low and high frequency noise. We can estimate the expected electrical noise by summing the noise contributions of the interface and pre-amplifier given in Eq. (27), the thermal noise of the equivalent input resistance and the 21 dB(A) capsule noise given in the datasheet [6] as

(32) $${\mathrm{Noise}}_{\text{(w), est.}}=\sqrt{{\mathrm{Noise}}_{\text{(w), pre-amplifier}}^{2}+\int_{200\;Hz}^{20\;kHz}4k_{B}T\left(r_{\text{in}}∥R_{\text{MK1}}\right)R_{w}{\left(f\right)}^{2}\;df+{\mathrm{Noise}}_{\text{(w), MK1}}^{2}}\;\;\text{yielding}$$

(33) $${\mathrm{Noise}}_{\text{(A), est.}}=3.0891\;µV\;\;\text{or}\;\;-110.20\;dB\text{V}\;\text{,}$$

for A-weighting, with $k_{B}$ as the Boltzman constant and T=300K as room temperature. Compared to the estimated electrical noise, the 2.15 dB increase is easily explained by imperfections in the isolation of the measurement chamber. Besides that, the results are as expected.

## 8 Conclusions

The pre-amplifier introduced in this article is cost-effective, easy to manufacture and demonstrates excellent performance, as established through extensive analytical calculations, SPICE simulation and empirical measurements. Its flat frequency response, low noise and minimal distortion make it an ideal pre-amplifier for integrating MEMS capsules in a pro-audio measurement setup compliant with the IEC 61938 P48 [5] microphone power standard.

To further enhance the design, an additional power rail for the input protection diodes $D_{1}$ to $D_{4}$ could be added to compensate for the diode forward drop voltage, which can cause clipping at higher input voltages. Moreover, the pronounced resonance frequency could be tackled by dampening the acoustic field with a special cloth in front of the capsule [25]. By reducing the diameter of the capsule PCB, the cloth could be clamped between the housing and the PCB, ensuring protection of the inlet vent and damping in the resonance region. For use in harsh environmental conditions, additional specifications such as temperature coefficient, pressure coefficient, influence of humidity, vibration sensitivity and magnetic field sensitivity should be tested [21].

With the integration of the IM73A135 [6] capsule, its pronounced resonance frequency slightly effects the otherwise flat frequency response. The cylindrical design facilitates easy mounting and serves as a drop-in replacement for conventional condenser microphones, while protecting the circuitry and capsule from environmental impacts. Additionally, the grounded aluminum housing acts as a shield and the balanced circuitry and transmission significantly reduces electromagnetic interference. The tested MEMS microphone exhibits good noise and low distortion characteristics, but there is still a gap to professional microphones. For measurements from 10 Hz to 10 kHz, the frequency response remains reasonably flat within a -3.9 dB to +4.9 dB range.

## CRediT authorship contribution statement

**F. Huber:** Writing – original draft, Visualization, Validation, Software, Resources, Project administration, Methodology, Investigation, Data curation, Conceptualization. **F. Toth:** Writing – review & editing, Supervision.

## Declaration of competing interest

The authors declare that they have no known competing financial interests or personal relationships that could have appeared to influence the work reported in this paper.
